# Homœopathy, Its Tenets and Tendencies, Theoretical, Theological, and Therapeutical

**Published:** 1854-03

**Authors:** 


### Art. IV.—HOMŒOPATHY, ITS TENETS AND TENDENCIES, THEORETICAL, THEOLOGICAL, AND THERAPEUTICAL.

By James Y. Simpson, M. D., F. R. S. E., *Professor of Midwifery, University of Edinburgh; and Physician-Accoucheur to the Queen for Scotland. President of the Medico-Chirurgical Society, and of the Obstetric Society; lately President of the Royal College of Physicians; formerly President of the Royal Medical Society, and of the Royal Physical Society, Edinburgh; Foreign member of the Imperial Academy of Medicine of France; member of the Society of Surgery of Paris; and of various Medical Societies in Stockholm, Copenhagen, Berlin, Ghent, &c., &c.* First American, from the Third Edinburgh Edition; Philadelphia: Lindsay and Blakiston, 1854.

Of all Medical heresies, Homœpathy has stood its ground for the greatest length of time, and exerted the most potent sway over the minds of men; and yet that it *is* a heresy no one can doubt who has looked into the subject. Our belief is, that the Homœopathists themselves have no faith in their medicines. We do not think it possible for any man, in his senses, to believe that the billioneth or decillioneth of a grain of oyster-shell, or camomile, or belladonna can exert the slightest possible effect upon the economy. Hahnemann, its founder, never believed it; in truth he had no faith in medicine, but thought sick people would be none the worse if left to themselves; and this is precisely where his system leaves them—with the *vis medicatrix naturæ.* He avowed that he gave medicines very seldom, though, he was in the habit of adding, he always prescribed *small powders.* He gave the "small powders" for the sake of keeping up in the patient's mind the belief that he was taking medicine. It was his observation, that most patients will recover by a simple regimen, and by the exercise of a boundless confidence in their medical attendants.

This is now believed, by all men who have thoroughly investigated the pretensions of Homœopathy, to be the principle upon which this most absurd system has so far succeeded. But our patients do not understand the secret. They see pulsatilla, acomite, and oyster-shell administered in infinitisimal doses and the subjects of the practice getting well. Homœopathy seems to them to cure the disease. How are they to learn where the fallacy lies? We answer, physicians must instruct them. The public must be informed through the medical profession. Let common sense be appealed to; let men understand what Homœopathy professes, and they will open their eyes. They will not discredit the cures which they have seen taking place under the lilliputian doses, but they will learn to laugh at the ridiculous assumption of the Homœopathist to have made them by his oyster-shell or belladonna globules. They will see that the cures were the work of nature, and not of the inane and negative practice resorted to. Most of the practitioners of this sect, and we incline to think all, fully understand this principle, and only apply Homœopathy in cases where the utility of more potent medication is not clearly apparent. They spread out the snare of their system "as a golden man-trap to catch credulous and trusting patients, and afterwards either openly or surreptitiously apply to them all the usual means employed in the armentarium of rational medicine." They give their remedies by the billionth of a grain in obscure or trivial cases, but by grains and scruples where experience has taught the efficacy of the article. They wish to be considered Homœopaths, but take care to drug their globules with active doses of the most powerful medicines. There was far more of knavery than of folly in the old inventor of this humbug, as his biographers have proved, and his followers, in this respect, have borne but too striking a resemblance to their master.

Homœopathy has become one of the facts of the times—not a "fixed fact," we think, but one which meets us in our professional rounds, and stares us in the face whenever we look out upon the world of physic. We cannot shun it nor blink it. It has got into high places, and ranks among its advocates men of science and note. We must, as a profession, deal with it, and, in the shortest practicable time, dispose of it. It has been exposed again, and again. Dr. SIMPSON, in the work before us, has exposed it once more, and his exposure is so thorough and complete that we have determined to treat our readers to some extracts from his eloquent and admirable book.

Some illustrations of HAHNEMANN's well known principle, *similia a similibus curantur*, are given by Dr. SIMPSON, which will probably be new to our readers, and we suspect, would startle the dupes of the Homœopathic practice. Dr. MURE, it seems, one of the ablest writers of this sect, has lately made the discovery that *itch* (the prolific source of our bodily ills, according to HAHNEMANN) is to be cured by small doses of lice, or lice-tea. Upon the same principle, "bugs in the thirtieth dilution," are recommended for curing the inflammation arising from bug-bites; the matter ejected by Cholera patients, after being "potentized," is to be swallowed for the cure of cholera; yellow-fever patients are to swallow the black vomit, duly prepared; the scales of scarlatina are used as a prophylactic against that disease; leucorrhea is cured by potentized leucorrheal matter, and the expectoration of phthisis is given to consumptive patients. Drs. TIETZE, SCHNAPPAUF, and other German Homœopaths with unpronouncable names, have lately treated small-pox patients with doses of the matter of small-pox and cow-pox. We wonder if human folly can descend to lower depths than these. When the deluded subjects of this practice come to understand the species of medication to which they have been submitting, can any one doubt that they will turn away from it with loathing and indignation?

But the Homœopaths are too shrewd to trust entirely to such remedies as these; they fully comprehend the method of inspiring their subjects with faith in their globules. The following extract contains the confession of one of these practitioners:—

"Patients who are skeptical of the truths of Homœopathy, from a love of variety, or a hundred other reasons, will consult you. As these persons are inclined to ridicule infinitesimal doses, it is sometimes highly useful to give them powerful doses of various highly concentrated medicines, in globules similar in appearance to all the rest, but consisting of morphia, strychnine, arsenic, corrosive sublimate, and such like; a few of these mingled with your sugar and starch globules will cause effects to be felt by the skeptic which will quickly overcome his disbelief; he generally makes an excellent patient, and often a good decoy-duck. Never scruple in paralytic cases to give strychnine largely, but never allow it to be supposed that you are giving more at a dose than the one-hundred-thousandth of a grain. This rule may be followed in other complaints with other very active drugs, such as croton oil; but this is one of our profoundest secrets, and must be kept so. Were it known, our wonder-working powers would be reduced in the estimation of the public and the regulars."

"It holds good," says HAHNEMANN, "and will continue to hold good as a Homœopathic therapeutic maxim, *not to be refuted by any experience in the world*, that the best dose of the properly selected remedy is always the very smallest one, as well for chronic as for acute diseases." His smallest dose, which he holds to be his most powerful, is the decillionth of a grain or of a drop of the drug, whatever the drug may be, strychnine, arsenic, or oyster-shell. What is this dose—*the decillionth of a grain?* Dr. SIMPSON attempts to give his readers some conception of it by the following illustration:—

"I have already stated that the earth is computed to contain, at the present time, some 900,000,000 human beings; and that if all these 900,000,000 had been called into existence when Adam was created—some 6000 years ago—and had lived up to the present time, and each of these 900,000,000 individuals had, when first called into existence, begun to swallow, and continued to swallow up to the present hour, without rest or cessation, night and day, a decillionth dose of a grain (say a grain of sulphur or quinine, or other drug,) such as Hahnemann recommends to be used, these 900,000,000 beings would not yet, during these past 6000 years, have finished one single grain of the medicine. Nay, if each of these 900,000,000 men, now 6000 years old, had swallowed, during *every* moment of their past existence, not a single globule, but *one million* of globules of Hahnemann's 30th dilution, they would not yet have finished a single grain, and would not finish it, working constantly every second at the same rate, for millions of centuries yet to come.

"Yet Hahneman and his followers calmly tell us, that—take two or three of these single decillionth doses of a single grain of their drugs, and with these infinitesimal doses of the requisite medicine you may cure any acute or chronic curable disease to which mankind is liable. And that one of these decillionths is truly the proper dose of the medicine to be used, is "a therapeutic maxim (says Hahnemann) not to be refuted by any experience in the world."

"To understand, if possible, the exiguity of "the best" dose of a homœopathic drug as thus laid down by Hahnemann, let us turn to anothor means of stating the infinitesimal amount of the dose. For this purpose let us suppose, merely for the sake of illustration, that the drug employed was commixed with a mass of sea-sand, as its vehicle or medium, instead of a mass of sugar or starch, such as is employed in forming homœopathic globules and powders.

"Most rational men, whether acquainted with medicine or not, would surely and justly look with perfect incredulity upon any physician who should maintain that *one* single grain of sulphur (to take it as an example) properly commixed with a heap of sand made by reducing to powder the stones, &c., employed in building and rearing all the houses in Edinburgh, or in London, or in Great Britain, would impart to each of the particles of the heap a sufficient amount of sulphur to enable it, if swallowed, to be a proper and sufficient dose for curing a man of itch. Or if some enthusiast were to spring up and aver that a single grain of phosphorus or of aconite, so infinitely divided, that an equal portion of one grain of these drugs distributed among *all* the particles of sand that lie on all the sea-shores of Great Britain, from Caithness to Cornwall, could change and medicate each one of these grains of sand, so that any dozen or two of them picked up along the coast of England or Scotland would, if swallowed, be certain to influence and cure a case of inflammation of the lungs, or of inflammatory fever, if given to men affected with these diseases, would the world not deem the asserter of this fact to be stating something beyond the bounds of mere extravagance and excentricity? And, if our supposed enthusiast were further coolly and deliberately to aver, that his single grain of phosphorus or aconite, if divided amongst all the grains of sand to be found along all the sea-shores of our world, would equally, if not more certainly, cure the same inflammation or fever, would he not be looked upon as a man outraging every principle of possible belief? But the Homœpaths aver infinitely more than this. Hahnemann and his followers, in using the 30th dilution of aconite, phosphorus, &c., aver that a *single grain* of any of them, distributed equally through a mass of sand as large as the bulk of the whole earth itself, or indeed through a mass of sand even millions of millions of times greater than the earth, would bestow the same healing property upon one and all of the particles of sand forming these immense masses, and that any ten or twenty of these medicated particles would form "the best" dose of the drug for the supposed case of pneumonia or fever.

"It has been computed that a cubic inch of sea-sand contains a million of particles of sand. The globe of our earth is reckoned be physical geographers to contain about four thousand millions of cubic feet, or nearly seven quadrillion cubic inches. If the whole mass of the earth from its surface to its centre were bruised down and converted into sea-sand, and a single grain of any homœopathic drug, as a grain of sulphur, or aconite, or phosphorus, were so commixed with the sand that each particle of sand had a proper relative share or allotment of this single grain, each of these particles would contain *more* sulphur, or aconite, or phosphorus, than the homœpaths use when they use the decillionth of a grain of sulphur, or phosphorus, or aconite, or any other of their drugs in the 30th dilution. It would require, in fact, a mass of sea-sand millions of times greater than the whole mass of the earth to reduce each particle of sand to a homœopathic decillionth.—Or, to state it otherwise, a decillion of particles of sea-sand would make a million of worlds or globes as large as the earth. The diameter of the earth is nearly 8000 miles; the diameter of the orbit of Neptune is nearly 6000 millions of miles; a vast difference. But even a world or sphere of sea-sand, with a diameter equal to that of the orbit of Neptune, would not have its particles reduced to a decillionth. In other words, each of the single particles in this vast mass would be larger than a decillionth of the whole; and the proper dose of a single grain of a drug divided equally among these decillionths would be one particle picked out of this inconceivable mass of sand.

"In order to impart to the human mind some faint, very faint, idea of the enormous distance of the stars, writers on astronomy have had recourse to a variety of illustrations. It has been computed that a cannon-ball, impelled with the greatest velocity which art can produce, would travel at the rate of 600 miles an hour; and flying at this rate, would require twenty years to pass from the earth to the sun, and about five millions of years ere it could reach the nearest fixed star. But the velocity of light is immensely greater. According to Struve's late investigations, it flies at the rate of 166,000 geographical miles, or nearly 200,000 English miles, in a second. Travelling at this enormous rate, a ray of light passes from the Moon to the earth in 1¼ second; from the Sun to the earth in 8 minutes; but would require about three years to travel from the fixed star *a Centauri* to the earth. Other stars, however, are vastly more distant The elder Herschel calculated that light, travelling at the enormous velocity we have stated, would require almost two millions of years to pass to the earth from the remotest luminous vapour reached by his forty feet reflecting telescope. And Professor Nichol imagines, from later observations with stronger instruments, that possibly there may be stars "situated so deep in space that the rays of light from them do not reach our earth until after travelling across intervening abysses during centuries whose number stuns the imagination." Possibly there may be some, he imagines, from which light would require to travel *thirty millions of years*, in order to reach our planet.

"Truly, such astronomical calculations do, as Professor Nichol observes, "stun the imagination" of man. They are as naught, however, in comparison with the ideas and computations of homœopathists. For one single grain of any homœopathic medicine, as sulphur, quinine, silex, &c., &c., &c., divided into decillionth globules of the 30th dilution, and arranged as twenty globules to the inch, would form a continuous line or string of globules reaching far, far beyond this inconceivable profundity in space. In other words, this grain of sulphur, &c., &c., &c., would, when divided into a decillion of homœopathic globules, make a line over which light, travelling at the rate of 200,000 miles in a second, would require above 1,000,000,000,000,000,000,000,000 years to pass along. Yet, aver Hahnemann and his followers, any one of these decillion globules of a single grain of the proper drug, picked out of this inconceivable length of line, would be found duly medicated for the treatment of all diseases, acute and chronic, and would form the most appropriate dose for their treatment. Truly the human imagination sometimes rapidly passes from the sublime to the ridiculous.

"A row of homœopathic globules, extending enough to form a string 25,000 miles long, and which consequently could encircle the earth, would be passed over by a ray of light in the eighth part of a second, or "in the twinkling of an eye." But it would require a ray of light to travel millions of millions of millions of years, before it could pass from one end to another of one single grain of sulphur, or any other homœopathic drug, laid out in homœopathic decillionth globules,—such as Hahnemann used and declared to be, both in acute and chronic diseases, the proper medical dose, "not to be refuted by any experience in the world." A continuous row of globules from Edinburgh to London—or, in other words, a row of them as long as an express railway train would take twelve hours to pass over, might, at first thought, appear extravagantly long, particularly when it was alleged that all of these numerous globules could have subdivided among them a single grain of a drug, so as to impart to *every* globule in the line the medicinal action of that drug. But how comparatively short is a 400 mile line of globules, to a line of them so extended, that light, travelling at the rate of 6,000,000,000,000 miles a year would take above 1000 sextillions of centuries to fly along it? And yet, as Hahnemann asserts, *one single grain* of a medicine distributed equally along this enormous line of globules, would invest *each* of the constituent globules with a power of curing diseases; and forms, in fact, *the* best and appropriate dose of each common drug.

"Some homœopaths, however, use their drugs not in GLOBULES or particles, such as the preceding illustrations refer to, but in tinctures or Solutions. Let us consider for a moment the tenuity of these homœopathic solutious or tinctures.

"Soon after the promulgation of Hahnemann's doctrines it was suggested that, "If the decillionth part of a grain have any efficacy, an ounce of medicine (Epsom salts) thrown into the Lake of Geneva would be sufficient to physic all the Calvinists of Switzerland."

"But more careful systematic calculculations have shown that this is stopping infinitely short of the truth; and that the 30th homœopathic dilution, recommended as we have seen by Hahnemann in all cases, is, in such a parallel, enormously understated, instead of over-stated. In fact, the 10th solution alone would, as Mr. Cap has shown, require for its proper solution, a body of water 500 times greater than the bulk of the Lake of Geneva; or a sea somewhat larger than the Gulf of Venice. To make the 11th solution, a quantity of water greater than the Mediterranean Sea or the German Ocean would be necessary. The 12th solution could scarcely be accomplished in a sea extending over the whole surface of the earth, and 500 fathoms in depth. And if the whole Solar System were buried in an ocean extending in depth from the Sun to Neptune, it would not form a sufficient fluid medium for adequately dissolving to the 30th dilution a common dose of any of the common medicines of the homœopathists. Yet the homœopathists allege, a few drops or sips of the proper medicine, properly dissolved in such enormous medicated seas and oceans, infallibly acts and cures; and that each drop would be found of "terrific potency," if the drug were duly mixed."

It is sometimes asked, how does it happen, if Homœopathy be such a nullity, that some eminent and able physicians have been made dupes to the delusion? We can only answer the question by pointing to the fact, that as eminent and able medical men have been carried away, in times past, by fallacies just as absurd. There were noted and learned physicians, half a century ago, who gave themselves up to the follies of Perkinism, who believed as firmly in the virtues of the metallic tractors as Dr. Henderson (of whose conversion we quoted from the work under notice an amusing account in our last number) does in the powers of the microscopic globules. Nay, have we not seen distinguished physicians, in our own day, stoutly insisting upon the truth of *clairvoyance?* The truth is, there is nothing too absurd to be embraced by minds of a certain stamp, and unfortunately not a few of these have been enlisted, in every age, in the service of medical quackery. Honest, conscientious men they have often been, but with minds incurably prone to humbug. With some mental infirmity, or else a self-esteem which led them to suppose themselves wiser than all other men, they have been the ready victims of impostures which the common sense of mankind at once detected. What at this moment is passing before our eyes? The whole credulous world led captive by "Spiritualism," by table-turning, and table-knocking, and in the mixed multitude of deluded followers a goodly proportion of medical men.

It is no argument for the truth of a system that it has respectable advocates, since as we have seen the most monstrous systems have found ardent and sincere adherents. But then, as has been admitted, there is that in Homœopathy which bears the semblance of truth, and which has kept it fresh and comparatively vigorous amid the decay of so many kindred fallacies. It inspires the sick with confidence, and leaves their cases to nature, which happily, is competent to the cure of most of them. But the delusion is on the decline. It is fast dying out in Germany, the land that gave it birth, and is losing ground in France and Great Britain. It will yield last in this country, but already we begin to perceive unmistakable signs of its decadence. This work of Dr. Simpson will hasten its dissolution, and for this reason we greatly desire to see it attain a wide circulation. We assure our readers that they could not procure a more *piquant* book, one abounding more in curious facts and instructive details, relating in any way to medicine, than it is. Let it be read and its incontrovertible facts given to the people.

